# Nursing Animosities

**Published:** 1882-07

**Authors:** 


### NURSING ANIMOSITIES.

How it warps the judgment, how it eats out the soul, how it kindles into fierce flame the worst passions of our nature, how it magnifies to mountain proportions, the veriest trifle which, in better and nobler moments, would have been passed over with an indifference which would not have given it a second thought. How mean it is too, to mope, and fume, and fret for half a night about a little grievance which, if it had been committed with the utmost deliberation, should have been passed over with a magnanimous forgetfulness. But when it is remembered that most frequently the offence is imaginary, or has arisen from thoughtlessness or inadvertence, the poor fretter becomes the object of our commisseration. Human life is too short, it is too holy a thing to be frittered away in cherishing animosities or in recording wrongs.

### SUGGESTIVE.

Fast men, convivial men, men who love to "sport," generally have a short life, although it is apparently a merry one, for the bottle is an inseparable companion; in some form or other, brandy or wine is an indispensable item. Twelve years ago, a sporting party in one of its convivial moods buried a bottle of liquor, with the agreement that the survivor of them all, should repair to the spot, dig it up, and drink it to their memories. A few days ago, the last man performed the sad task. Only twelve years ago, and of all that merry company a single man remains! A temperate life, a life of religion expressing itself in daily good doing would have told a very different story.

